# A Mixed Methods Research Study of Parental Perception of Physical Activity and Quality of Life of Children Under Home Lock Down in the COVID-19 Pandemic

**DOI:** 10.3389/fpsyg.2021.649481

**Published:** 2021-03-15

**Authors:** Gabriela López-Aymes, María de los Dolores Valadez, Elena Rodríguez-Naveiras, Doris Castellanos-Simons, Triana Aguirre, África Borges

**Affiliations:** ^1^Transdisciplinary Research Center in Psychology, Autonomous University of the State of Morelos, Cuernavaca, Mexico; ^2^Institute of Psychology and Special Education, Department of Applied Psychology, University Center for Health Sciences, University of Guadalajara, Guadalajara, Mexico; ^3^Facultad de Ciencias Sociales, Universidad Europea de Canarias, La Orotava, Spain; ^4^Faculty of Psychology and Speech Therapy, Department of Clinical Psychology, Psychobiology and Methodology, University of La Laguna, Santa Cruz de Tenerife, Spain

**Keywords:** home lock down, physical activity, quality of life, pandemic, mixed methods research

## Abstract

Household confinement due to the rapid spread of the pandemic caused by COVID-19 has brought very significant changes, such as the forced stay-at-home of children due to the closure of schools. This has meant drastic changes in the organization of daily life and restrictions on their activities, including exercise, which could affect the quality of life of the children due to its importance. In order to study the relationship between physical activity and psychological well-being of minors, a study has been carried out with Mixed Methods Research, combining survey methodology with transversal design with qualitative methodology using discourse analysis. A total of 234 parents of minors in Spain and several Spanish-speaking countries in America participated. The instrument was a questionnaire in Google Forms, which included the Kidscreen-27 quality of life scale. The results show significant differences in both the type of physical activity and its frequency due to age, and differences in parents’ perception of whether their children’s physical activity levels were sufficient or not, both on the health, mood and school subscales, and in the categorization of open responses referring to concerns due to the pandemic, analyzed with the ALCESTE technique. The relationship between physical activity of children and adolescents and quality of life is clearly concluded.

## Introduction

At the end of December 2019, the first evidence appeared in Wuhan, China, that a new lethal viral disease had emerged, for which no vaccine or specific medication was available. In March the disease became a pandemic and a large majority of countries, either with specific regulations or through recommendations to the population, established confinement and social distance as the possible solution to prevent further spread of the disease, to avoid saturation of hospitals and curb the lethality of the virus. On March 10th, the global situation with regard to COVID-19 was 113,702 confirmed cases (4,125 new) and 4,012 deaths (203 new) (World Health Organization [WHO], 2020a). On December 29, the number of confirmed cases worldwide was to over 79 million, with a cumulative death toll of over 1.7 million (World Health Organization [WHO], 2020b).

In the field of Psychology, theoretical formulations have been made to explain the reasons why COVID-19 evolved so rapidly and was so widely spread. Urzúa et al. (2020) point out three factors: (a) illusory optimism; (b) inadequate perception of absence of contingencies produced by the population’s behavior; (c) optimistic risk perception. Vera-Villarroel (2020) has stated that physical and mental health are closely linked, and explains the expansion of the pandemic based on three psychological processes: cognitive, with the population having irrational beliefs about the disease and illusory optimism; emotional, with feelings such as fear, stress and anger; behavioral, with exposure and risk behaviors. The author points out that these factors must be considered in the intervention to save lives.

Several studies have shown the risk that social isolation caused by the pandemic implies not only for the most exposed groups (health workers), but also for the mental health of the general population. Problems of anxiety (Chew et al., 2020; Holmes et al., 2020; Wang and Zhao, 2020), stress and psychological distress have been reported, both during and even after the biodisaster (Liu D. et al., 2020). Along the same lines, the narrative review conducted by Huarcaya-Victoria (2020) points out three types of problems for the general population: health anxiety, depression and stress. Rajkumar (2020) groups the problems derived from the pandemic into four sections: general population, health workers, vulnerable people and therapeutic strategies and interventions. The author emphasizes the need to study the effect of the situation generated by the pandemic on children and adolescents.

A particularly vulnerable group in this whole situation is children and adolescents. Although results in children for Coronavirus-19 disease are still inconsistent. Changes produced in their environment since COVID-19, such as the restrictions that home isolation and not being able to access the main areas of socialization (Socías et al., 2020), with risks such as stress from both them and their parents, since COVID-19 can cause psychological alterations in children such as those caused by other stressors (Espada et al., 2020; Socías et al., 2020).

Certain factors can have effects not only during confinement but also afterward, such as the disappearance of healthy habits like attending classes, which have been replaced by unhealthy behaviors, such as sedentary lifestyles, inappropriate diets, excessive use of screens which can produce, in addition to weight gain, physical problems (Brazendale et al., 2017; Wu et al., 2017). From this follows the importance of understanding the effects that a wide variety of personal and contextual factors (Holgado-Tello et al., 2010) can have on children and adolescents and their interaction in the way they experience physical activity and sports during the pandemic situation. Other risks that have been highlighted, depending on age, include substance abuse, accommodation issues and overcrowding and change and disruption of social networks (Holmes et al., 2020). It is expected that, after confinement, in most cases these problems will disappear (Barlett et al., 2020), although some may persist after the situation generated by the pandemic has passed (Espada et al., 2020). Space restrictions and not being able to go outside are especially important in childhood for the proper development of playing, which is essential for its maturation process (García-Serrano and García-Fernández, 2015).

In view of the difficult situation experienced, the population has been provided with recommendations, some of which have been generated by institutions to support their citizens (Socías et al., 2020). These guidelines have many points in common: maintaining routines, being active, supporting minors, carrying out social activities, in short, maintaining a normal life in safety (Liu J. J. et al., 2020). The support of parents is important, who can strengthen family ties and meet the needs of children through appropriate parenting styles (Wang et al., 2020). The need for physical exercise is also stressed (Holmes et al., 2020; Mera-Mamián et al., 2020; Romero et al., 2020). Physical exercise plays a relevant role both on a physical level (Vidarte Claros et al., 2011) and in mental processes (Ramírez et al., 2004; Zhou et al., 2020) as well as on a psychological level (Berger and Motl, 2000; Biddle and Mutrie, 2001; Tessier et al., 2007; Anderson and Brice, 2011). In particular, there is clear evidence of the contribution of physical activity to psychological well-being (Molina-García et al., 2007; Jiménez et al., 2008; Romero et al., 2009; Fernández Ozcorta et al., 2015).

The relationship between physical activity and well-being linked to the quality of life has been the subject of multiple investigations in recent years, which have also emphasized its influence on the general health of the various sectors of the population (Schwartzmann, 2003; Bize et al., 2007; Anokye et al., 2012). In particular, different studies have highlighted the association between high levels of physical activity, or the practice of sports, and the quality of life in children and adolescents (Anokye et al., 2012; Marker et al., 2018; Luna et al., 2019).

Likewise, recent reviews of studies on interventions focused on the promotion of sports practices and their impact on issues such as mental health, self-esteem, anxiety levels, and perception of well-being in children and adolescents, underline the benefits of this kind of activities for the general health of this population in particular, showing that physical-sport education pilot programs might promoted significant improvements in specific indicators of subjective well-being and emotional intelligence of participating adolescents’ groups (Bermejo-Cantarero et al., 2017; Luna et al., 2019).

The lack of physical activity is a widely reported public health problem (Bermejo-Cantarero et al., 2017). For this reason, evaluation that focuses on the relationships between physical activity and health-related quality of life is an important focus of research in this field. On the other hand, there is little research aimed at exploring parents’ knowledge and perceptions of their children’s physical activity, their ideas about its importance and impact on the way they experience diverse dimensions of a stressful life (Gallego-Méndez et al., 2020; Spinelli et al., 2020; Yarımkaya and Esentürk, 2020) particularly during the Coronavirus outbreak. Exploring these issues, including the different perspectives of persons involved in families’ life (Izquierdo-Sotorrío et al., 2016), could help provide recommendations and support programs for parents to guide their children’s physical activity.

In the case of children and adolescents, physical activity has important benefits: it promotes growth and enhances both physical development (Rosa et al., 2018) and psychomotor, cognitive and social development, and generally favors all body systems: metabolism of carbohydrates and lipids, control of blood pressure, decreases the risk of type 2 diabetes and improves body composition (Camargo Lemos and Ortiz Dallos, 2010).

Physical activity also favors psychological factors: it helps to build a balanced self-concept and improves self-perception, mood, self-image, physical self-concept, perception of health and life satisfaction, and intellectual function (Camargo Lemos and Ortiz Dallos, 2010; Reigal-Garrido et al., 2012; Rosa, 2015).

The home quarantine imposed by the COVID-19 may make physical activity more difficult, and as we have seen in the studies reviewed, this leads to a decline in the quality of life of children and adolescents. Quality of life (QoL) is understood as personal satisfaction (or dissatisfaction) with the cultural or intellectual conditions in which an individual lives. Health is one of the domains of quality of life, this domain comprises not only physical health but also psychological health, as well as the interaction that people have with others and with the community (Ravens-Sieberer et al., 2005). For this research, we are interested in reviewing the quality of life, based on the assessment of the well-being perceived by parents.

Given that the collection of high quality data is a priority in order to understand the psychological effects that the quarantine may have produced in the population, and that there is an urgent need to discover, analyze and evaluate the psychological interventions that could alleviate the problems generated and minimize the risks that could occur in the mental health of society (Holmes et al., 2020), the aim of this research is to analyze parents’ perceptions of their children’s quality of life in relation to observed physical activity in the conditions of staying in the housing due to the pandemic situation due to the COVID-19. It hypothesizes the existence of greater quality of life perceived by parents who consider their children to be sufficiently physically active.

In this sense, we try to find out if there is any difference in quality of life between children of different ages and sex in the conditions of staying in the housing due to the pandemic situation due to the COVID-19 as perceived by mothers and fathers. In addition, it is investigated whether the characteristics of the housing (the space) conditioned the perception of the parents about their children doing more or less physical activity, and whether there are differences between the age and the type of physical exercise done. It is also interesting to know the relationship between the level of physical activity and psychological well-being.

## Materials and Methods

### Methodology and Design

This is a non-experimental design. Mixed methodology was used (Mixed Methods Research, MMR; Johnson and Onwuegbuzie, 2004; Denscombe, 2008). The data was collected through a cross-sectional design with survey methodology, using an *ex post* facto design, and there are open questions that allow a qualitative analysis.

To determine the differences in physical activity, three independent variables were considered: age (children, adolescents), sex (male, female), as well as a third variable, grouping parents according to their opinion about the physical activity developed by their children in confinement (sufficient, insufficient). The dependent variables used has been the different scales that make up the KIDSCREEN test, which therefore requires multivariate analysis.

### Participants

A total of 234 participants responded to the survey. The average age was 42.82 (SD = 7.10), with a range between 24 and 65. More mothers (203) than fathers (30) participated, and only one of the informants was guardian of the minors, relative in charge of the child. Table 1 presents the data regarding age (values corresponding to the percentiles 25, 50, 75, and over 75) and educational level. The procedure for selecting the sample was one of convenience.

**TABLE 1:** Parents’ age and educational level.

		Parental educational level			Total
		Without studies	High School	University studies	
Age	37 or less	0	24	23	47
	38–42	1	10	49	60
	43–47	0	24	42	66
	48 or plus	0	11	50	61
Total		1	69	164	234

Parents and caregivers were asked to think of one of their children when answering the questionnaires. In this way, for the data analysis, they were grouped by the ages of the children, the largest group being children between 8 and 11 years old, 125 (52 female) and 109 adolescents between 12 and 17 (54 female).

The countries of origin of the participants were mainly Spain (134, 57.3%), and Mexico (86, 36.9%) and others American countries (Panamá, Colombia, Argentina, and Chile; 13, 5.8%).

Most families (230, 98.3%) reported not having been victims of the coronavirus. Only four families had a confirmed patient in the family unit, and in four other cases there was a suspicion that a family member had the disease.

In the questionnaire, a question was included about family and housing conditions. Most of the sample lived in the same dwelling with up to four family members (167, 71.4%), while it was less frequent for the family size to be greater than four (67, 28.6%). The average number of rooms, discounting common services, such as kitchen, living room and bathroom, was 3 (113 of the participants, 48.3%), with a range between 1 and 10 rooms. Most of the dwellings have at least one exterior space (177 of the participants, 75.6%).

### Instruments

A questionnaire was designed to obtain data on parents’ perceptions of their children’s physical activity, some specific data on the type of housing during their child’s confinement. This questionnaire consists of 18 questions (15 closed, 3 open-ended) distributed in the following categories: (1) descriptive data of the participants (6 items); (2) family and housing conditions (5 items); (3) issues related to the situation produced by the COVID-19 pandemic (3 items); (4) complaints and needs caused by the situation produced by the COVID-19 pandemic (4 items) (see Supplementary Data Sheet 1). At the end of the questionnaire it was mentioned that if they wanted to ask for the results of the research they could leave their e-mail. All questions were marked as mandatory in the Google form, so there was no room for incomplete or missing data.

For the HRQoL measure, the Kidscreen-27 Parent Questionnaire (Ravens-Sieberer et al., 2005). Spanish version was used, once the authorization for its use in this study was requested and obtained. This is a questionnaire that assesses health-related quality of life. This questionnaire was used because it provides a parameter to contrast the perception of psychological and health well-being in the child population with the physical activity observed by the parents. It consists of 27 items, which are answered in a Likert-type scale of five alternatives (from nothing to very much), structured in five scales: physical activity (4 items), mood (7 items), family life (7 items), friends (4 items), and school (4 items), and a single question about your child’s general state of health in the last week. The test is filled in by parents, for children and adolescents between the ages of 8 and 18. The original authors (Ravens-Sieberer et al., 2005) offer evidence of the factorial validity of the test and its reliability in all the subscales of the test, in terms of internal consistency, with the total Cronbach’s Alpha value equal to 0.82. With our data, a similar Alpha of 0.831 has been obtained.

### Procedure

The questionnaires were assembled in electronic format with the Google Forms application. It was sent out by email and through social networks (Whatsapp, Facebook, and Twitter) to contacts in different educational associations, using the snowball technique. It was sent during the month of May 2020 (it can be defined as the first period of confinement). Only one of both parents was asked to answer the questionnaire with one of their children in mind (in case they have two or more), and who was in the age range of 8–17 years. The time required to fill in the questionnaire was 15 to 20 min.

At the time of data collection, all participants (regardless of country) were in the same conditions of confinement, leaving the home only for essential activities, with restrictions on going to school, physical activities or recreation outside the home.

As far as ethical aspects are concerned, the Commission on Ethics in Research and Animal Welfare of the University of La Laguna (CEIBA) was asked to authorize the study, which was granted (Registration Number: CEIBA2020-0396). In the questionnaire, the corresponding information for the participants was set out in the Organic Law 3/2018, of December 5th, on Personal Data Protection and guarantee of digital rights (BOE, 2018), guaranteeing the anonymity and confidentiality of the data.

### Data Analysis

The relationship between parental consideration of physical activity sufficiency and having or not having outdoor space in the home was calculated using the V of Cramer.

To check the absence of univariate outliers, we used Tukey’s test that takes as reference the difference in interquartile range, considering a slight outlier at 1.5 times this distance, and extreme when it is at three times that distance. To determine the existence of multivariate outliers, the Mahalanobis distance was calculated.

Regarding quality of life, it was analyzed in two ways taking three independent variables: age, sex and parents’ assessment. Since the quality of life variable, measured by Kidscreen, is split into several scales, it requires a multivariate approach, so three MANOVAs were carried out, one according to each independent variable studied. All quantitative analyses were conducted with the SPPS program, v.21.

For the qualitative analysis, the phenomenological discourse analysis method was used, which identifies the meanings of language, through lexical analysis using the ALCESTE software (in French: *Analyse des Lexèmes Coocurrents dans les Enoncés Simples d’un Texte*) (Reinert, 2003). This program facilitates the analysis of linguistic materials that generally arise in social research, such as answers to open-ended questions in questionnaires, in-depth interviews or answers based on projective techniques (De Alba, 2004). The ALCESTE methodology consists of three stages: the construction of the data matrix, the classification of the context units (statements) and the description of the classes (Gil et al., 1994). The methodology focuses on the statistical distribution of word succession, taking into account only the simultaneous presence of several words in the same statement. In this way, classes are identified as semantic fields, represented in trees or dendograms. In the ALCESTE method, the initial text is broken down into elementary contextual units (ECUs), which approximately match the size of a sentence.

The statistical analysis, although limited to explain in detail the meaning of a text, allows the elaboration of a “cartography” of the lexical worlds chosen by the speaker to express himself and, therefore, of the reference systems from which he constructs his way of seeing reality (Gil et al., 1994; Reinert, 2003).

## Results

### Quantitative Analysis

#### Physical Activity

In order to know if there is a relation between the participant’s perception of the sufficiency or not of the physical activity developed by his or her child and the space dedicated to exercises, these variables were analyzed, considering in the household conditions whether there was no outdoor space to carry out activities or if, on the contrary, there was. The results are shown in Table 2. There is significant dependence between both variables (V of Cramer = 0.146; *p* = 0.026).

**TABLE 2:** Perception of adequacy of physical activity and space for it.

	Outdoor space for physical activity		
Physical activity	No	Yes	Total
Insufficient	44	108	152
Sufficient	13	69	82
Total	57	177	234

#### Elimination of Outsiders

Eleven extreme univariate cases were eliminated and none multivariate by Mahalanobis distance, with the criterion of probabilities less than 0.001.

#### Psychological Well-Being by Age and Sex

Most parents consider their child’s health to be excellent (88, 39.5%) or very good (114, 51.1%), while only 21 (9.4%) rate it as “fair.”

The group was divided into two ages: from 8 to 11 (children) and 12 and older (adolescents). Table 3 shows the descriptive statistics.

**TABLE 3:** Age and sex level descriptive statistics.

Scale	Age	Female			Male		
		Mean	SD	N	Mean	SD	N
Health	Children	8.10	3.56	49	7.66	3.83	71
	Adolescents	6.56	3.86	52	6.33	4.01	51
Mood	Children	14.45	2.10	49	14.31	2.17	71
	Adolescents	15.55	2.36	52	13.61	2.33	51
Family	Children	20.02	3.90	49	20.11	4.43	71
	Adolescents	19.77	4.47	52	20.65	4.48	51
Friends	Children	6.82	4.43	49	7.10	4.63	71
	Adolescents	9.53	3.60	52	10.13	3.51	51
School	Children	11.41	2.54	49	10.83	3.41	71
	Adolescents	11.27	2.84	52	10.35	3.27	51

To know if there are differences by sex and age, a MANOVA was calculated, which was for sex (Wilk’s λ = 0.949, *F*_5_._215_ = 2.3, *p* = 0.046, Partial η^2^ = 0.051), and for age (Wilk’s λ = 0.843, *F*_5_._215_ = 8.034, *p* = 0.001, Partial η^2^ = 0.157) nor for interaction (Wilk’s λ = 0.982, *F*_5_._215_ = 0.796, *p* = 0.554, Partial η^2^ = 0.018). Individual ANOVA results are only significant for the variable age in the health scale (*F*_1_,_219_ = 7.692, *p* = 0.006, Partial η^2^ = 0.034), with a small effect size and in the friend one (*F*_1_,_219_ = 28.421, *p* < 0.001, Partial η^2^ = 0.115), with a large effect size.

#### Physical Activity and Well-Being

In order to assess whether the children developed adequate physical activity, the parents were asked whether they considered it sufficient or insufficient. A total of 146 considered it to be insufficient and 77 sufficient. The informants were divided into two groups according to this variable and it was analyzed whether there were significant differences in their assessment of the psychological well-being of the children. Table 4 presents the mean values and standard deviations of each welfare scale.

**TABLE 4:** Descriptive statistics of physical activity and well-being.

	Physical activity	Mean	Standard Deviation	N
Health	Insufficient	5.86	3.15	146
	Sufficient	9.73	3.85	77
Mood	Insufficient	14.20	2.32	146
	Sufficient	14.31	2.15	77
Family	Insufficient	20.03	4.21	146
	Sufficient	20.32	4.55	77
Friends	Insufficient	8.39	4.40	146
	Sufficient	8.26	4.30	77
School	Insufficient	11.37	3.03	146
	Sufficient	11.20	3.15	77

The result of the MANOVA was significant (Wilk’s λ = 0.743, *F*_5_,_217_ = 15.001, *p* < 0.001, Partial η^2^ = 0.257). Individual ANOVA results are only significant for the health scale (*F*_1_,_223_ = 64.821, *p* < 0.001, Partial η^2^ = 0.227), with a large effect size.

### Qualitative Analysis

In order to find out the perceptions that families have regarding different aspects of stay-at-home confinement, both required by law and recommended, four open-ended questions were analyzed by ALCESTE, separating into two samples parents who considered that their children were getting enough exercise and those who thought it was insufficient: (a) Explain why you say you have sufficient or insufficient physical activity; (b) How did your child live it?; and (c) What or who does your child miss?

### Analysis of the Question “Explain Why You Have Sufficient or Insufficient Physical Activity”

**FIGURE 1:**
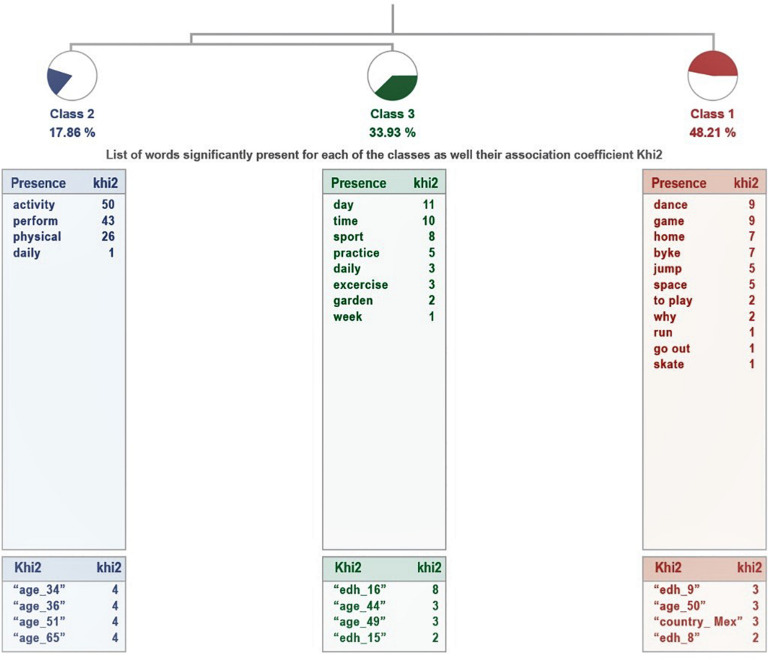
Dendogram corresponding to the question “Explain why you have sufficient physical activity.”

The analysis of ALCESTE, for the group of parents who consider that their children have sufficient physical activity (see Figure 1), the results are grouped into three classes, which explain 66% of textual units. The first class is linked to the link between the second and third classes. The most representative class is 1, as it groups the largest number of EUs. The details of the analysis, in terms of class name, UCEs grouped and percentage involved, most representative word and examples, are presented in Table 5.

**TABLE 5:** Analysis of the question “Explain why you have sufficient or insufficient physical activity.”

Class		ECU	%	Word
**They consider their children’s activity to be sufficient**				
1 Name	*Movement and play*	27	48.21	Dancing
Phrases	Plays a lot at home.			
	Because he likes to ride his bike, he plays hide-and-seek and tries to always run up and down			
	Right here in the house he climbs the couches, goes up and down the stairs and starts dancing.			
2 Name	*Sports practice*	10	17.86	Activity
Phrases	He has performed physical activity every day			
	We do physical activities together at home			
	In addition, since we have moderated the children’s outings, we have encouraged them to do activities on the move and not just walk			
3 Name	*Daily physical activity*	19	33.93	Day
Phrases	Every day practice sport online			
	Practice two and a half hours a day of conditioning and classical ballet			
	We do some sport every day			
**They consider their children’s activity to be insufficient**				
1 Name	*Impossibility to leave*	20	17.9	Lack
Phrases	Lack of time to go out			
	We still need to go out more			
	Because we can’t go out on the street			
2 Name	*Little exercise*	34	29.06	Sport
Phrases	She should do more sport but it is very vague, even the blackmail do it for me that I have a lot of weight and not even			
	He used to do a lot more. Playground, soccer team, friends.			
	He does a 20 minutes’ routine of varied exercises. He could do more, but he prefers to spend more time with his friends			
3 Name	*Lack of space for physical activity*	21	17.95	Sufficient
Phrases	My daughters are active and need the outdoors			
	Not enough space at home			
	In the house there is not enough space to release the energy generated every day			
4 Name	*Absence of physical activity*	18	15.38	Physical
Phrases	No physical activity			
	Does not do physical activities			
	Does not do too much physical activity			
5 Name	*Decrease in usual exercise routine*	14	11.97	Week
Phrases	From working out several times a week to sporadic exercises without a daily routine			
	She practiced several hours of exercise a day, which is impossible today.			
	I can’t motivate her to exercise more. Sometimes we go for a walk, but not daily.			
6 Name	*Disinterest in physical activity*	10	8.55	Done
Phrases	Does not exercise as she is used to or has not been able to go to her pools			
	Does not exercise as she is used to or has not been able to go to her pools			
	Has done online classes of ballet, very motivated but with complicated logistics, and some of zumba but was not very motivated with this			
	She has not done any exercise, neither moderate nor continuous. She started physical activity on Mondays and ended up being very			
	sore. She does not want to activate herself much. Only from time to time following just dance videos			

**FIGURE 2:**
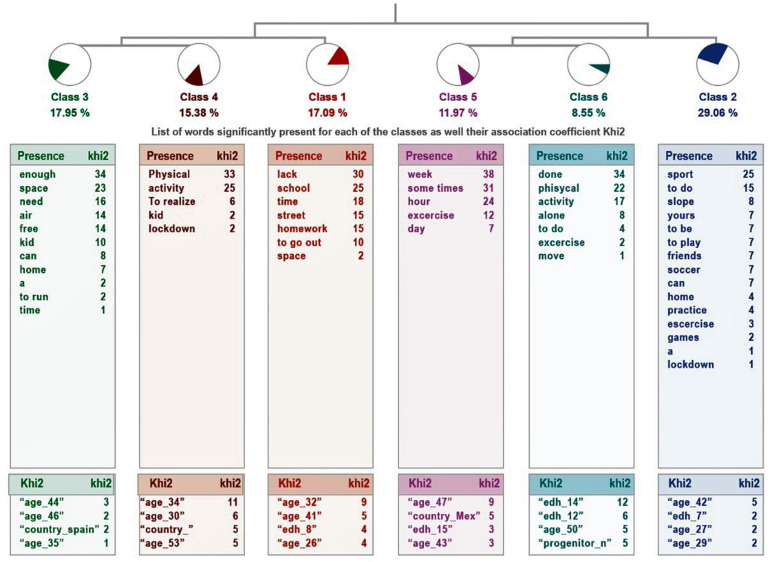
Dendogram corresponding to the question “Explain why you have insufficient physical activity.”

The reasons given by parents for considering that their children could not get enough physical activity are more dispersed, as they have been grouped into six clases (see Figure 2). In this case, there are two groupings: on the one hand, class 2 connects with the union of classes 5 and 6, while class 1 connects with the link between classes 3 and 4. Classes 1, 5, and 6 are related to the impossibility of doing either exercise or sports that they did before the pandemic, while the difficulties of the other set of classes go in the direction of lack of space and the need to go outside.

### Analysis of the Question “How Did Your Child Live Not Being Able to go Out on the Street?”

**FIGURE 3:**
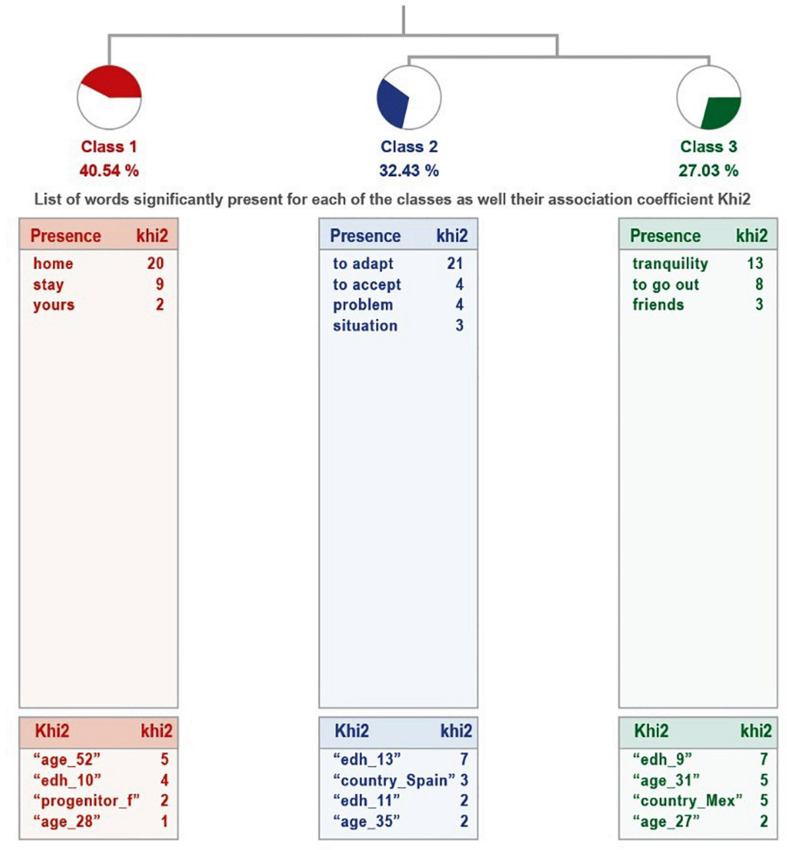
Dendogram of the question “How did your child live not being able to go outside?” Sufficient physical activity.

The analysis of the group that considers that their son or daughter has had enough activity explains 51% of the text corpus. The dendogram is shown in Figure 3.

**FIGURE 4:**
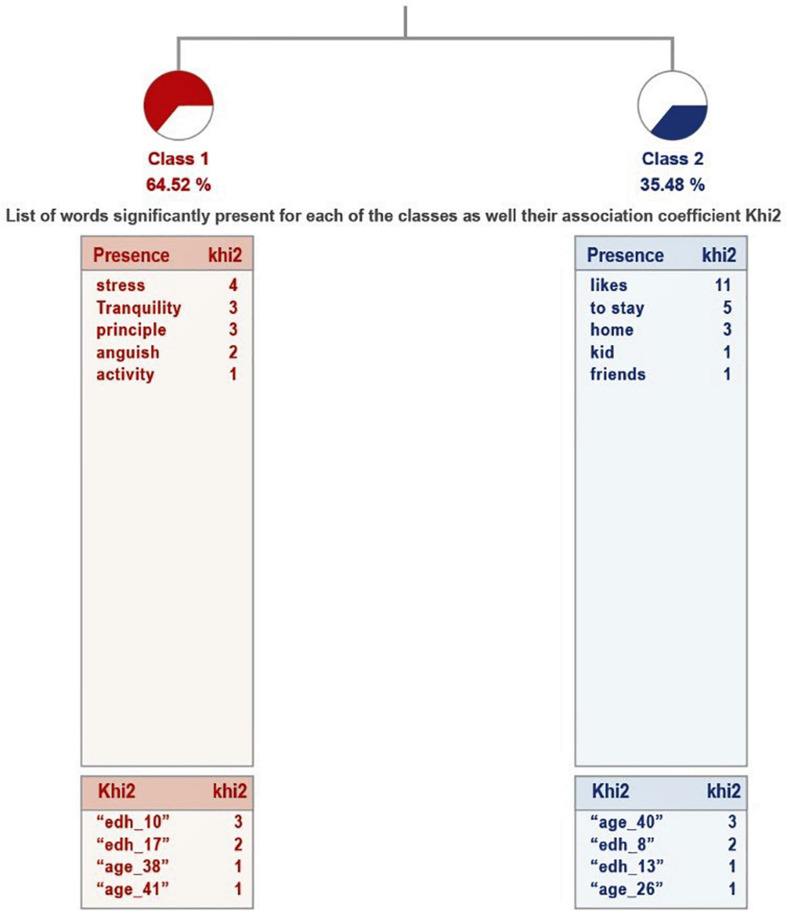
Dendogram of the question: “How did your child live not being able to go outside?” Insufficient physical activity.

On the other hand, in the group of parents who consider the activity performed by their children insufficient, although it explains only 27% of the corpus, extracting only two classes (Figure 4).

Table 6 shows the detail of the classes, in terms of their name, number of UCEs they group, percentage of the corpus they explain and the most representative word, as well as representative examples of each class. In both groups, a distinction is made between positive aspects, of being at home, or pointing out some kind of problem.

**TABLE 6:** Information from the analysis to the question “How did your child live not being able to go out on the street?”

Class		ECU	%	Word
**They consider their children’s activity to be sufficient**				
1 Name	*Pleased to be home*	15	40.54	Home
Phrases	phenomenal likes to stay at home			
	well it didn’t seem wrong to stay at home			
	happy to skip school and stay home			
2 Name	*Acceptance to stay home*	12	32.43	Adaptation
Phrases	Well he had no problem accepting the situation			
	Well accepted the situation and adapted to it			
	Well without problems accepting the situation and adapting			
3 Name	*With tranquility*	16	32.65	Quiet
Phrases	Quietly did not want to go out and was entertained			
	Normal, no problem quietly for not being able to leave			
	Calm had no problem not to go out			
**They consider their children’s activity to be insufficient**				
1 Name	*Stress*	20	64.52	Stress
Phrases	My son suffered a lot of anxiety and stress at first over-all.			
	Quiet at first, but when schoolwork began with stress and anguish			
	Calm at first, a lot of stress because of the lack of physical activity, the lack of contact with children his age, the confinement, etc.			
2 Name	*Being comfortable at home*	11	35.48	Comfortable
Phrases	Well, he likes to be at home, he is not very sociable.			
	likes to be at home			
	Says she likes to be at home but feels she lacks physical activity and refuses to do it.			

### Analysis of the Question “What or Who Does Your Child Miss?”

**FIGURE 5:**
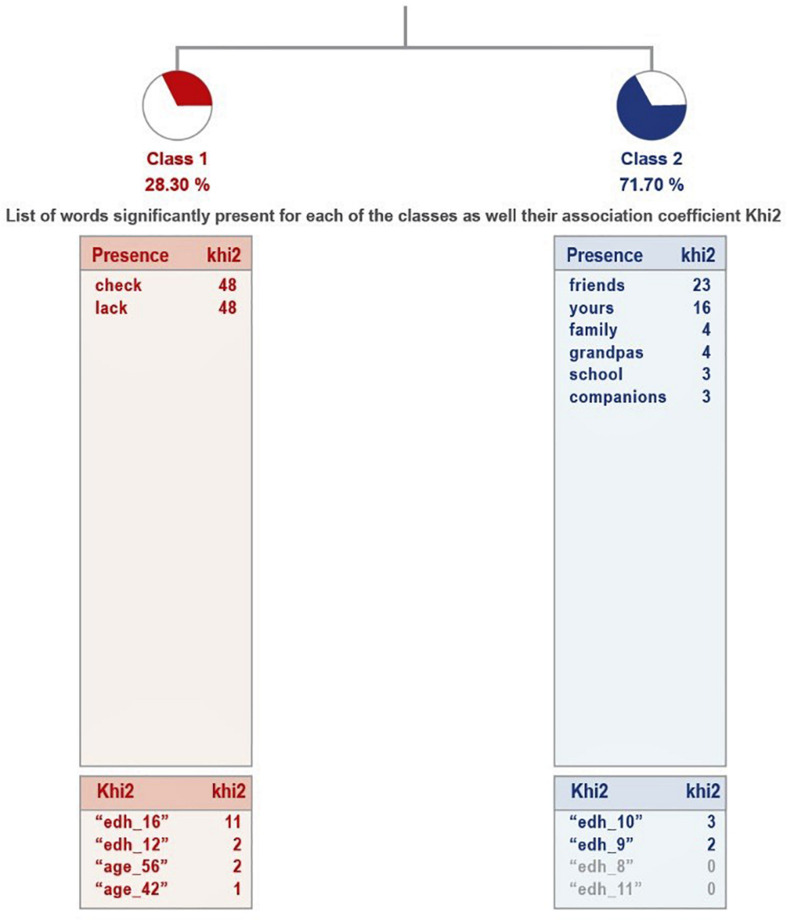
Dendogram of the answers: What or who does your child miss? Sufficient physical activity.

The analysis of this question, for the group of parents who consider that the physical activity developed by their son or daughter is sufficient, gives two classes, which explain 65% of the textual units, that is, an average relevance of the treatment (see Figure 5). These are two antagonistic classes: the second is the one that groups the most textual units (71.70%), where it is clear that the child misses both the extended family and the people in his or her school environment. The first class includes those who responded that they have not missed anything or anyone and is quite homogeneous: they do not miss anyone (see Table 7).

**TABLE 7:** Information from the analysis to the question “What or who does your child miss?”

Class		ECU	%	Word
**They consider their children’s activity to be sufficient**				
1 Name	*He doesn’t miss anything or anyone*	15	28.30	Miss
Phrases	He doesn’t miss			
2 Name	*Missing extended family and friends*	17	71.70	Friends
Phrases	Family and friends of the school			
	Grandparents, friends, outings, trips, school			
	Grandparents, relatives, friends and teachers.			
**They consider their children’s activity to be insufficient**				
1 Name	*He doesn’t miss anything or anyone*	19	17.27	Miss
Phrases	He doesn’t miss			
2 Name	*She misses going out to play*	20	18.18	To go out
Phrases	To be able to go and play basketball or soccer, to be able to be with friends the trainings play chess go out			
	go out and play			
3 Name	*Missing family and friends*	13	11.82	Family
Phrases	Family, friends.			
	Friends, family and outings			
	Family and friends			
4 Name	*School context*	17	15.45	Mates
Phrases	The school and his classmates, as well as his teacher.			
	School and classmates.			
	The school and his classmates!			
5 Name	*He/she misses her grandparents and friends*	24	21.82	Grandparents
Phrases	To her friends, her grandmothers and her guardian			
	To his grandmother, the contact with his friends			
	To his friends			
6 Name	*Missing the extended family*	17	15.45	Their
Phrases	To his mother the grandparents the cousins.			
	Dad, grandparents, cousins, and friends			
	His cousins and his grandparents			

In the case of parents who feel that their son or daughter does not get enough physical activity, there are six classes, with a grouping of classes on a ladder: from class 1 to 4 are connected individually, linking class 5 with 6.

**FIGURE 6:**
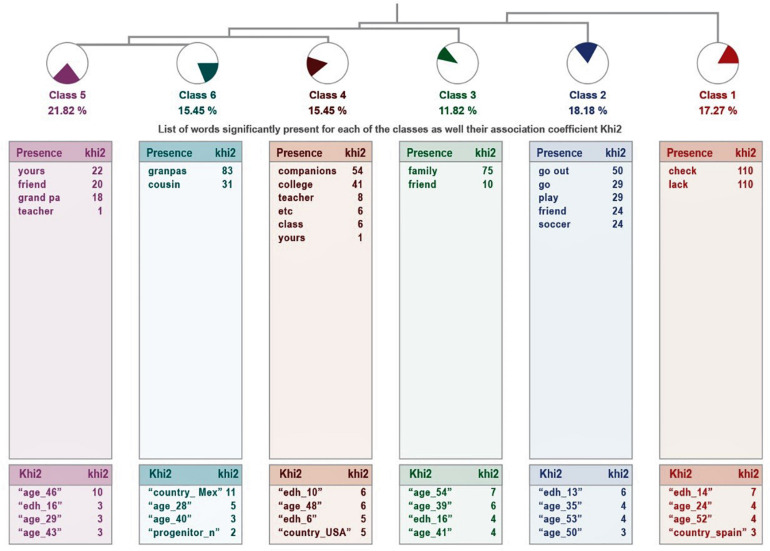
Dendogram of the answers: “What or who does your child miss?” Insufficient physical activity.

It explains 72% of the textual units, which means that the relevance of the treatment is high. They are presented in Figure 6.

## Discussion

The first thing to note is that the data collection was done in the months of April and early May, at the time of the most severe confinement, as in Spain, Mexico, Panama, and Argentina (BBC, 2020). It is true that the regulatory conditions regarding confinement have differed in the countries where the participants in this research live, in some cases, such as Spain, being obligatory by the State of Alarm, while in other countries governments strongly recommended avoiding going out and staying at home. This has meant 24-hour family life, with parents having to telework and children being taught online. The possibilities of exercising under these conditions have been very limited, which can have important effects on the psychological well-being of the minors.

As far as the health of their children is concerned, a large majority consider it to be good or very good. Furthermore, taking into account the five scales of quality of life in relation to age levels (children and adolescents), parents value the health of their children more the younger they are. In contrast, differences in contact with friends score higher for adolescents.

Parents’ perceptions of their children’s quality of life significant differences are observed with respect to sex at the global level, which is not maintained in the scales separately, but they do differ by age on two of the instrument’s scales: health, where younger children score higher, and friends, with the opposite result, as would be expected: adolescents score significantly higher on this scale.

Physical activity is conditioned by the type of housing. The results show that when there is no outdoor space to develop physical activity, parents find that exercise performed by their children is insufficient more often.

The objective of this study, to establish whether there is a relationship between physical activity and psychological quality of life in the conditions of confinement at home from the parents’ perspective, has been clearly corroborated, both in quantitative and qualitative analyses, finding differences between the two established groups of participants: those who considered that their children could develop sufficient physical exercise versus those who thought it was insufficient. Divergences are shown in both groups at the quantitative and qualitative levels.

With respect to the quality of life instrument, there are significant differences between the overall scores of the two groups; however, significant differences are only found in the health scale; when parents consider that the physical activity developed by their children is insufficient, lower scores are obtained in that scale. These results support the hypothesis of a positive relationship between quality of life and physical activity.

The differences found between the two groups of parents (those who consider their children’s physical activity sufficient and those who do not) in the quantitative analyses are also verified in the qualitative ones. The second group of parents shows more dispersion in the open responses given, as well as greater concern.

Thus, in the first open question analyzed qualitatively, “*Explain why you have sufficient or insufficient physical activity*,” the discourse of some parents differs significantly, as it is obvious, since the reasons they give for the physical activity done by their children being sufficient must be differentiated from those who consider it to be insufficient. In the latter, two perspectives are clearly distinguished in the two branches that appear in the dendogram: lack of space or impossibility of doing the exercise they would like to do. Moreover, it also confirms what has already been commented, that is, how there is a relationship between physical space and the facilities of households to exercise is related to the satisfaction or dissatisfaction with the physical activity performed by their children.

The second question, centered on their child’s experiences of not being able to go out, parents who feel their children have enough physical activity, report that their children experienced the lock down positively. On the other hand, in the other group there is a division of opinions: one part considers that their children lived the lock down without problems, but others think that their children lived it with stress, being this last one the most representative class. It confirms again a greater decline in the quality of life of their children for this group.

Finally, in the question relating to whether their child misses something or someone, there is greater variability among the children whose parents consider they do not have enough activity, since the answers are grouped together in one more class, where there is content where school life is missing.

The limitations of this work are about convenience samples, since there is no guarantee of absence of selection bias. However, having included several countries, all of them with a significant restriction on going out of the house, it gives indications of cross validity. This unusual development of the pandemic has evened out the differences between nations in a common struggle against an unprecedented biological crisis.

As far as the uncertainty of living under what has come to be called the new normality, together with the certainty that the threat of the pandemic is not over and that outbreaks, more or less virulent, may occur, it is particularly relevant to carry out research on mental health and psychological well-being, in order to be able to foresee more precisely the actions to be taken, knowing the dangers involved. Holmes et al. (2020) point out how important it is to accumulate experience based on the evidence that has provided the lessons learned so that those in power can coordinate measures that will damage the lives of citizens as little as possible, especially those who are most vulnerable. In this regard, since children are a vulnerable sector of the population, knowledge of their reactions and how they have been affected is particularly relevant. For future research, this could also include children’s self-report, comparing their perception with their mothers and fathers’s (Izquierdo-Sotorrío et al., 2016). As a general recommendation in the light of the data collected, emphasizing the importance of exercise in guaranteeing the psychological well-being of minors is vital and must be conveyed to the population.

## Data Availability Statement

The raw data supporting the conclusions of this article will be made available by the authors, without undue reservation.

## Ethics Statement

The studies involving human participants were reviewed and approved by The University of La Laguna’s Ethics Committee of Research and Animal Welfare has approved this research (Registration Number: CEIBA2020-0396). The patients/participants provided their written informed consent to participate in this study.

## Author Contributions

ÁB, GL-A, MV, and DC-S had participated in theoretical review. ÁB, ER-N, GL-A, DC-S, and MV had participated in research design and instrument. ÁB had participated in the data analysis. ÁB, ER-N, GL-A, DC-S, and MV had participated in discussion. ÁB, ER-N, GL-A, DC-S, MV, and TA had participated in the study planning, writing, and revision of the article. All authors contributed to the article and approved the submitted version.

## Conflict of Interest

The authors declare that the research was conducted in the absence of any commercial or financial relationships that could be construed as a potential conflict of interest.
