# Supplementary material for: A Mixed Methods Research Study of Parental Perception of Physical Activity and Quality of Life of Children Under Home Lock Down in the COVID-19 Pandemic
Source: Front Psychol. 2021 Mar 15;12:649481. doi: 10.3389/fpsyg.2021.649481 (PMC8005529; doi:10.3389/fpsyg.2021.649481)
Supplement: Supplementary file 1 [file Data_Sheet_1.docx]

**Annex 1. QUESTIONNAIRE CONTENTS**

| **1. Descriptive issues of the sample** |
| --- |
| 1. Person completing the questionnaire 2. Participant's age 3. Educational level of the participant 4. Country of residence 5. Child's age 6. Sex of child |
| **2. Family and housing issues** |
| 1. Cases of coronavirus in the immediate family 2. How many people are living in your home at this time 3. How many rooms does your house have without counting the living room, kitchen and bathroom/s? 4. Does your home have space to exercise? 5. Type of space where to exercise |
| **3. Pandemic issues** |
| 1. In your country, has there been a time during the coronavirus crisis when it was forbidden, or strongly recommended, for minors to go out on the streets? 2. How did you experience it? 3. How your child experienced it |
| **4. Complaints and needs** |
| 1. Does your child complain or protest about the seclusion/healthy distance situation? 2. What is his/her complaint? 3. Does your child miss anything or anyone? 4. Who does he/she miss? |
